# On the Diseases of the Jaws, with a Brief Outline of Their Anatomy, and a Description of the Operations for Their Extirpation and Amputation, with Cases and Illustrations

**Published:** 1845-07

**Authors:** 


### ART. XVIII.

*On the Diseases of the Jaws, with a brief Outline of their Anatomy, and a description of the Operations for their Extirpation and Amputation, with Cases and Illustrations.* By R. O'SHAUGHNESSY, F.R.C.S.E. &c. &c. and Superintendent of the Gurrunhattah Dispensary.—*Calcutta,* 1844. 8vo.

IT always gives us particular pleasure to notice the efforts of our countrymen in the remoter provinces of our mighty empire, to extend the knowledge of medical and surgical science among their immediate neighbours. A book written by *one of ourselves,* has an influence very superior to that of even a better book coming from a distance. Hence we have ever encouraged the literary exertions of our friends in India; and it is but justice to admit that these have claimed encouragement not merely from their relative but from their actual value. The present thoroughly practical little work of Mr. O'Shaughnessy (brother we presume of the eminent Indian physician of the same name) is richly entitled to the same meed of praise. It is dedicated "to the students and graduates of the Medical College of Bengal," as a manual for their guidance in proceeding to perform any of the bold operations required for the removal of the diseases described in it. The first thirty pages are devoted to a brief account of those forms of disease which most frequently give rise to the necessity for operations on the jaws, as fibro-cartilaginous tumours, osteo-sarcoma, spina ventosa, exostosis and epulis, and the results of common inflammation, exfoliation, abscess, &c.; and ten pages more are assigned to the anatomy of the parts. To both these portions we think some extension might have been given with advantage. The remaining sixty pages are occupied by a description of the mode of operating for the removal of the upper and of the lower jaw, and by the detail of five cases in which one or other of these operations was successfully performed, illustrated by lithographic drawings of four of the tumours before removal.

The following is the plan of operation recommended by the author in amputating the superior maxillary bone; and we are sure that the eminent surgeons named in our extract will be the first to encourage a free examination of their chirurgical proceedings. Mr. Liston and Mr. Fergusson can well afford to be criticised.

"The patient is to be placed in a strong arm-chair, with his head resting against the breast of an assistant, or on a crutch attached to the back of a chair. A second assistant stands at the patient's side prepared to make pressure on the carotid artery, should it be necessary to do so in the course of the operation. The operator then takes his place in front of the patient, and should the extent of the disease require the removal of the whole of the malar and maxillary bones, he makes an incision commencing at the zygomatic arch, and terminating in the angle of the mouth. This incision should be first drawn over the zygoma as far as the malar eminence, then downwards over the surface of the tumour, to within half an inch of the angle of the mouth and into the cavity of the mouth, through the centre of the commissure of the lips, the knife being guided by the fore and middle finger of one hand placed for the purpose in the mouth. By dissecting this flap upwards, the whole of the attachments of the tumour may be laid bare; by detaching the upper lip, and ala of the nose, the nasal process and hard palate are exposed. The zygomatic process is to be freed from the temporal fascia superiorly, and from the masseter muscle inferiorly at the point to be divided by the nippers. The orbital process is next exposed by raising the conjunctiva of the eye with the inferior oblique muscle. All these incisions and dissections except the last should be made with rapidity, as there are no parts of any importance endangered before arriving at the orbit. The cheek is next dissected downwards and backwards for a little way, and then the hard attachments are severed with the bone nippers in the following order: The zygomatic arch is first to be cut through; the malar bone is next to be separated from its connexion with the external angular process of the os frontis, by cutting backwards into the spheno-maxillary fissure, taking care to guide the forceps with the fore-finger, so as to save the eye from injury. The nasal process must now be cut through by inserting one blade of the nippers into the nostril, and the other into the angle of the orbit, from this the floor of the orbit may be divided by cutting it across with a strong knife to the spheno-maxillary fissure. An incisor tooth or two if necessary, is next to be extracted, and the palate process as far back as its junction with the palate bone, cut through with the nippers keeping close to the tumour, in order not to remove more healthy bone in this situation than is absolutely necessary. The whole of the hard attachments being now divided, the tumour is found to be moveable, and in general slight pressure is found sufficient to displace it; when the knife is again resumed, and the external pterygoid muscle posteriorly, and the masseter muscle anteriorly, and the mucous membrane at the back of the mouth and cheek cut through, and the tumour removed.

"No matter how large the tumour, and how great the consequent distension of the cheek may have been, I recommend most strongly that no portion of the skin of the face if healthy be cut away. It almost invariably contracts to very nearly if not completely its natural dimensions, and if any of it has been removed with the tumour, no matter how small that portion may be, the want of skin enough is much more likely to be complained of when the cure is completed, than of there being too much if the whole has been left."

It will be seen that the incision through the integuments employed by Mr. O'Shaughnessy is less extensive than those recommended by Mr. Liston and Mr. Fergusson; on this point our author makes the following observations:

"Mr. Liston's directions for forming the flap are, to make an incision over the external angular process of the frontal bone, to be carried downwards through the cheek to the corner of the mouth. A second incision is made along and down the zygoma, falling into the other. Then the knife is pushed through the integuments to the nasal process of the maxilla, the cartilage of the ala is detached from the bone, and the lip is cut through in the mesial line.

"With great deference to so high an authority on all points of operative surgery, and particularly with reference to this operation I venture to differ with Mr. Liston as to the necessity of making three incisions through the integuments of the face, at least in the generality of cases, viz., one from the os frontis to the mouth, a second meeting this at right angles over the malar bone, and a third along the side of the nose and through the upper lip. I think the single incision described above, will be found to answer all the purposes proposed. By it the zygoma can be exposed with ease, a little dissection upwards will lay bare the frontal and malar bones, and with the cheek, the ala of the nose and upper lip may be raised, and the alveolar and nasal processes exposed to the fullest extent. Of course cases occasionally present themselves requiring additional incisions, but I think when they can be avoided, (and I believe in the majority of cases they are not necessary,) it will be found of advantage not to make them.

"Mr. Lizars and Mr. Fergusson recommend that the saw should be applied to all the bony processes before attempting to cut through them with the nippers; but this I think quite unnecessary, as with the latter instrument they may be divided with perfect ease and a smoothness, and certainly with far greater despatch, and less pain to the patient, than by using the saw whose action can with great difficulty be confined to the hard parts, and in fact its use is only necessary in the case before pointed out, viz., to divide the malar process where the malar bone may be saved."

In all the cases detailed the tumour was of a large size. In the first, a fibro-cartilaginous tumour of the upper jaw, the mass removed weighed four pounds; and in the last, a case of osteo-sarcoma of the lower jaw, the tumour was as large as a child's head, necessitating the amputation of all the lower jaw, excepting the left ramus. All the patients did well.

---

